# Population genomics of virulence genes of *Plasmodium falciparum* in clinical isolates from Uganda

**DOI:** 10.1038/s41598-017-11814-9

**Published:** 2017-09-18

**Authors:** Shazia Ruybal-Pesántez, Kathryn E. Tiedje, Gerry Tonkin-Hill, Thomas S. Rask, Moses R. Kamya, Bryan Greenhouse, Grant Dorsey, Michael F. Duffy, Karen P. Day

**Affiliations:** 1 School of BioSciences, Bio21 Institute/University of Melbourne, Melbourne, Australia; 2 Department of Microbiology, New York University, New York, USA; 3 Walter and Eliza Hall Institute, Melbourne, Australia; 4 School of Medicine, Makerere University College of Health Sciences, Kampala, Uganda; 5 Department of Medicine, University of California, San Francisco, USA

**Keywords:** Genetic variation, Malaria

## Abstract

*Plasmodium falciparum* causes a spectrum of malarial disease from asymptomatic to uncomplicated through to severe. Investigations of parasite virulence have associated the expression of distinct variants of the major surface antigen of the blood stages known as *Pf* EMP1 encoded by up to 60 *var* genes per genome. Looking at the population genomics of *var* genes in cases of uncomplicated malaria, we set out to determine if there was any evidence of a selective sweep of specific *var* genes or clonal epidemic structure related to the incidence of uncomplicated disease in children. By sequencing the conserved DBLα domain of *var* genes from six sentinel sites in Uganda we found that the parasites causing uncomplicated *P. falciparum* disease in children were highly diverse and that every child had a unique *var* DBLα repertoire. Despite extensive *var* DBLα diversity and minimal overlap between repertoires, specific DBLα types and groups were conserved at the population level across Uganda. This pattern was the same regardless of the geographic distance or malaria transmission intensity. These data lead us to propose that any parasite can cause uncomplicated malarial disease and that these diverse parasite repertoires are composed of both upsA and non-upsA *var* gene groups.

## Introduction

Children living in many regions of Africa are at risk of febrile episodes of malaria until they eventually acquire immunity that protects them against the development of clinical disease. This immunity is non-sterilizing and occurs after repeated exposure to *Plasmodium* spp^1^.

*Plasmodium falciparum* Erythrocyte Membrane Protein 1 (*Pf* EMP1) has been proposed as the major variant surface antigen (VSA) of the most virulent malaria parasite known as *P. falciparum*. This highly polymorphic antigen is encoded by up to 60 diverse *var* genes present in each parasite genome^2–4^. Differential expression of these genes leads to switching of *Pf* EMP1 variants in a single cell^5–7^. This mechanism of clonal antigenic variation occurring in a hierarchical manner allows the parasite to evade the host immune response to *Pf* EMP1 thereby establishing a chronic infection. High levels of diversity have been observed in the *var* gene repertoire in a single parasite^8–11^ as well as in populations^12–15^. Importantly, *Pf* EMP1 is also a virulence factor as it mediates adhesion of infected erythrocytes to host endothelial cells and expression of specific variants is associated with the pathogenesis of uncomplicated, severe and placental malaria (reviewed in e.g. ref.^16^).

Characterization of the structure of *var* genes has revealed a conserved domain architecture consisting of an N-terminal segment (NTS), followed by multiple Duffy-binding like (DBL) and cysteine-rich interdomain region (CIDR). This is despite the sequences being highly polymorphic^8,11^. *Var* genes can be further classified into four main sub-groups based on semi-conserved upstream promoter sequences (ups): groups A (upsA), B (upsB), C (upsC), and E (upsE) with group E consisting of only *var2csa*, a gene associated with placental malaria^9,10,17,18^. Recombination events occur mostly between *var* genes from the same group, generating high sequence diversity while still preserving the *var* domain architecture^19^. Despite this considerable diversity, comparisons to distantly related primate parasites (e.g. *P. reichenowi*) have shown that balancing selection has maintained ancient *var* sequence fragments over millions of years^20^. Where malaria transmission is high, multiple-clone *P. falciparum* infections are common^21,22^ leading to increased rates of meiotic recombination resulting in extensive repertoire diversity^23,24^. Moreover, high levels of mitotic recombination have been observed within cloned laboratory lines^25^. Whether this mitotic diversification process occurs as frequently in nature remains an open question.

Evidence from case-control studies have shown that group A *var* genes are preferentially expressed in children with severe and/or cerebral malaria, group B *var* genes are associated with clinical malaria (uncomplicated and severe) and cerebral malaria, and group C transcripts are present in larger proportions in children with chronic asymptomatic malaria infections^26–34^. Sequencing of seven *P. falciparum* genomes has shown that all parasites have the group A and B *var* genes^11^ so expression of these genes rather than the presence of these genes *per se* is associated with disease severity.

Population genetic studies of *var* genes have focused on sequencing the highly conserved DBLα domain and demonstrated by limited sampling that extensive diversity in DBLα sequences and repertoires exist in endemic areas of South America, Africa and Papua New Guinea^12–14,35,36^. Recently, we investigated the population structure of *var* DBLα sequences in parasites from asymptomatic children from an area of high malaria transmission in Gabon, West Africa^15^. Strikingly, in this first study of deep sampling of *var* genes in the asymptomatic reservoir in Africa we found that every parasite isolate had a distinct *var* DBLα repertoire with minimal sharing of DBLα types among 200 isolates. The absence of parasite genomes with high sharing of types was consistent with immune selection structuring a large effective parasite population size, as defined by DBLα diversity, into repertoires as different as possible in a transmission system favoring outcrossing. The case was made that these data provide evidence for a novel type of “strain structure” in a system where recombination is the key driver of diversification of the major genes under immune selection. These important observations regarding the *var* population genomics of asymptomatic infections in children lead us to ask the question: what is the population structure of *var* genes in uncomplicated malaria cases of *P. falciparum* in an area of high transmission in Africa? We wanted to explore whether they exhibit the same population structure or whether there is epidemic transmission of parasites with related repertoires of *var* genes causing uncomplicated malaria. Moreover, we wanted to explore the population structure of different groups of *var* genes (i.e., upsA and upsB/upsC) to see if they had distinct patterns of genome evolution under variable conditions of transmission.

Specifically, our experiments describe the *var* DBLα diversity of the parasite population causing uncomplicated *P. falciparum* cases in children under five. We present results for six sentinel health sites in Uganda and demonstrate that *P. falciparum* parasites causing uncomplicated cases in children were highly diverse with distinct *var* DBLα repertoires despite varying levels of transmission intensity across Uganda. In our study we found no evidence of epidemic expansion or clonal propagation of *P. falciparum* parasites with highly related *var* DBLα repertoires in the population.

These data led us to propose that any parasite can cause uncomplicated disease. Furthermore, these highly diverse parasite repertoires contain both upsA and non-upsA *var* gene groups in proportions expected from whole genome sequencing^11^.

## Results

### Diversity and frequency distribution of DBLα types

DBLα amplicons were pooled and sequenced from 517 of the 600 isolates from the six sentinel sites.

A total of 51,401 DBLα sequence reads were obtained from the 517 isolates and clustered based on average linkage using a 96% sequence identity threshold resulting in 21,156 unique DBLα sequences, or DBLα types. Sixteen (3%) isolates with less than 20 DBLα types were excluded from analyses due to limited DBLα type counts. From the 501 isolates utilized for this analysis, a total of 50,624 DBLα sequence reads were obtained (mean = 8,437; range = 4,347–10,950 per site) and we observed a total of 21,134 DBLα types (mean = 5,766; range = 3,012–7,366 DBLα types per sentinel site) (Table 1).

**Table 1:** *Var* DBLα sampling by sentinel study site.

Study Sites	No. isolates sampled	Total *var* DBLα sequences sampled	Mean no. DBLα types per isolate^a^ (range)	Total no. DBLα types	No. DBLα types seen once^b^ (%)	No. upsA DBLα types (%)^c^
Kanungu	64	4,374	68 (22–195)	3,012	2,299 (76.3)	369 (12.3)
Jinja	89	8,713	98 (48–208)	6,108	4,816 (78.9)	697 (11.4)
Kyenjojo	83	6,567	79 (40–165)	4,421	3,339 (75.5)	481 (10.9)
Arua	97	10,950	113 (50–262)	7,366	5,723 (77.7)	838 (11.4)
Tororo	91	10,726	118 (20–211)	7,266	5,565 (76.6)	732 (10.1)
Apac	77	9,294	121 (46–254)	6,424	5,043 (78.5)	771 (12.0)
All Sites	501	50,624	101 (20–262)	21,134	12,493 (59.1)	1,624 (7.7)

^a^Calculated by the following formula: *total var DBLα sequences sampled/no. isolates sampled* in that particular site. ^b^Refers to the DBLα types that were seen only once in all isolates from a given sentinel site based on the frequency distribution of DBLα types in that particular site (see Fig. 1). ^c^Calculated by the following formula: *total upsA DBLα types/total no. DBLα types* in that particular site.

**Figure 1:**
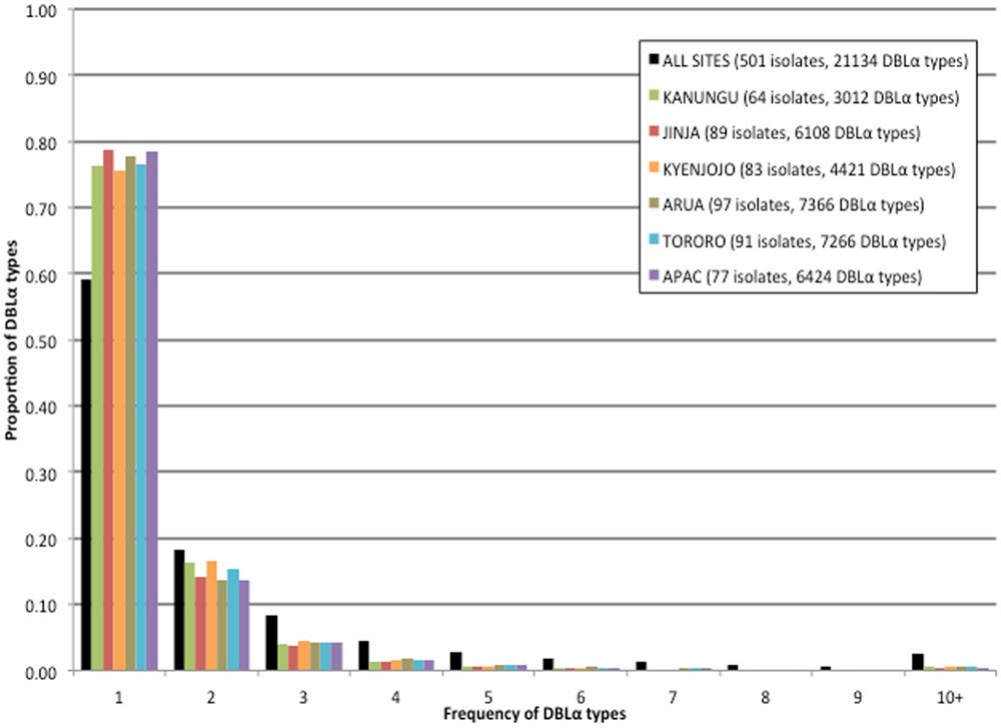
Proportion of DBLα types appearing 1 to 10 or more times within and among all sentinel sites.

Within and among the sentinel Ugandan sites sampled the distribution of the DBLα types showed a similar pattern of abundance (Fig. 1). The majority of the DBLα types were rare and only found in one isolate per site, whereas very few DBLα types were seen more than once per site (Fig. 1). A total of 12,493 DBLα types (59.1%) were seen only once in all sentinel sites where the minimum and maximum number of times a type was seen was 1 and 288, respectively (Table 1, Fig. 1). Within each sentinel site the proportion of DBLα types seen once ranged from 75.5% to 78.9% (Table 1, Fig. 1). Interestingly, among all sites 8,641 DBLα types (40.9%) were seen more than once with 536 DBLα types (2.5%) seen in 10 or more isolates (Fig. 1). Of the 536 types seen in at least 10 isolates, 374 were observed in isolates from Gabon^15^ with 121 seen at a frequency above 2.5%. Note that due to the reoccurrence of some DBLα types among the sites, the total number of observed DBLα types in all sites is lower than the cumulative sum of DBLα types observed in each site (Table 1).

We further classified the 21,134 DBLα types into upsA or upsB/upsC by translating the DBLα types and then characterizing them as upsA or upsB/upsC according to their domain class (see Methods and Supplementary Text 1). The DBLα type sequence groups were distributed as expected from whole genome sequencing (e.g. ref.^11^) with upsA representing 7.7% (1,624 DBLα types) and upsB/upsC representing 92.3% (19,510 DBLα types) of the total number of DBLα types sequenced in Uganda (Table 1). The distribution of the DBLα types showed type-specific patterns of abundance when the upsA and upsB/upsC DBLα types were considered separately (Supplementary Figure S1). When comparing the proportion of upsA to upsB/upsC DBLα types across all sentinel sites, although less abundant in the total population, specific upsA DBLα types were significantly more likely to be observed in 10 or more isolates (i.e. more conserved in the population) than the upsB/upsC DBLα types (*p* < 0.001). This pattern of increased abundance of the upsA DBLα types being more conserved was only statistically significant within the high transmission sites of Arua (*p* = 0.026), Tororo (*p* < 0.001), and Apac (*p* < 0.001). For the low to moderate transmission sites (Kanungu, Jinja, and Kyenjojo) there was no significant repeat occurrence of specific upsA DBLα types. It is important to note that the conserved upsA DBLα types represented a minority (13.1%) of the total upsA DBLα types observed.

### Depth of DBLα sampling

Using previously published methods, the cumulative diversity curves were utilized to measure the depth of DBLα sampling in each of the six Ugandan sentinel sites and among all sites^12,13^. A curve that approaches an asymptote and levels off indicates a well-sampled population. Even though a large dataset of DBLα types was obtained for clinical cases, the cumulative diversity curves did not plateau for any of the sentinel sites, indicating the population was not sampled deeply enough to observe all types (Supplementary Figure S2)^12,13^. The cumulative diversity curve for upsA DBLα types appears to be leveling off (i.e., approaching saturation) in comparison to the curve for upsB/upsC DBLα types, indicating an approximation of the total upsA DBLα types in the total Ugandan population (Supplementary Figure S3). DBLα richness was estimated by calculating Chao2, ICE and Jackknife2 statistics, which are designed to capture the extent of missing data^37–42^. We estimated a total of 41,345 DBLα types in all sentinel sites (95% CI = 40,376–42,363) by Chao2 statistics, 44,092 types by ICE statistics and 42,207 types by Jackknife2 statistics (Supplementary Table S1). Using the Chao2 richness estimates, the proportion of DBLα types sampled was estimated to be relatively high in all the Ugandan sentinel sites (51.1%) (Supplementary Table S1). Within each sentinel site, the proportion of total DBLα types sampled by Chao2 richness estimates ranged from a minimum of 30.9% to a maximum of 37.1%, with a mean of 33.7% (Supplementary Table S1).

### Relatedness of DBLα repertoires within isolates

**Figure 2:**
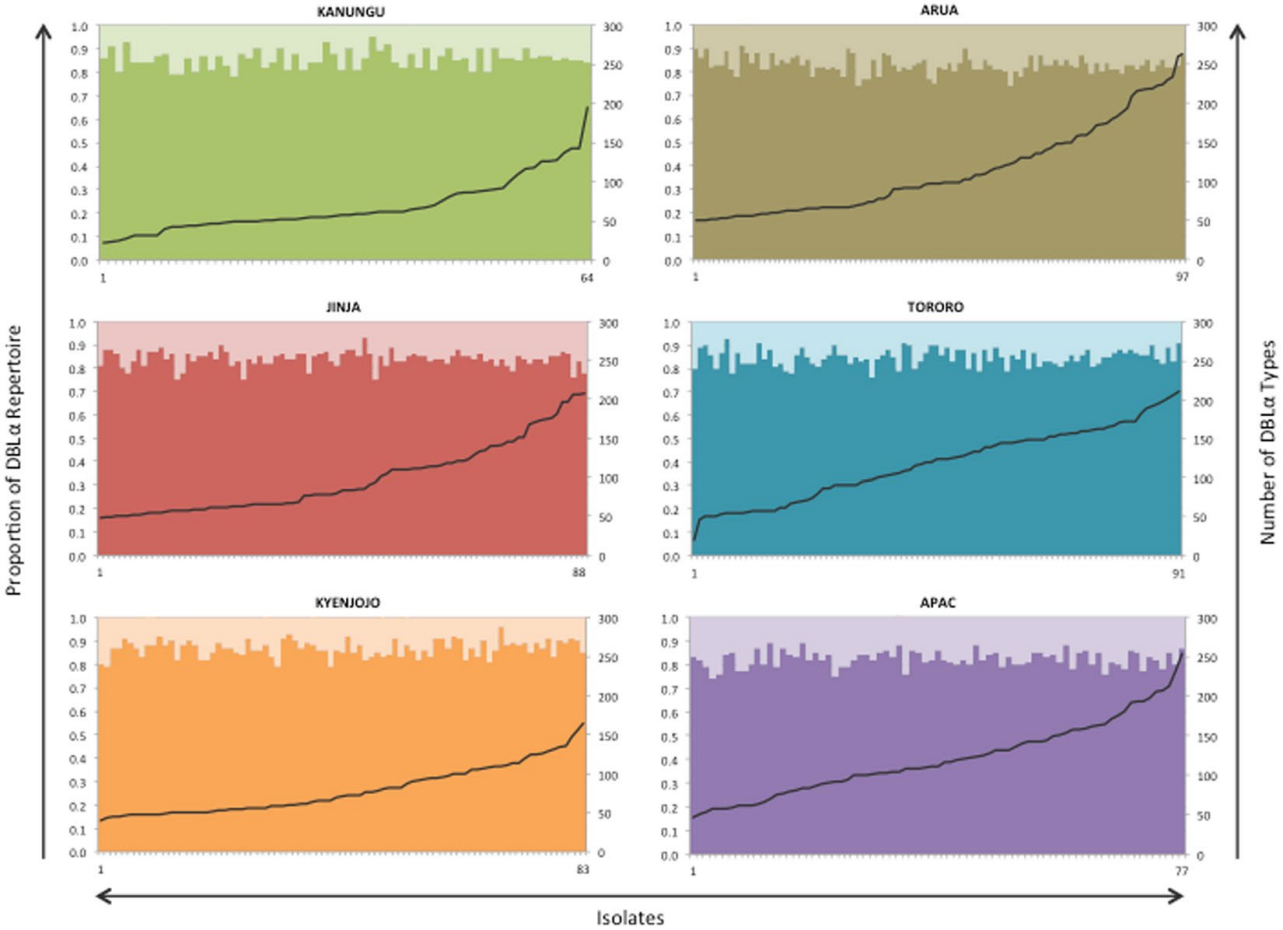
Schematic presentation of the major sequence group (upsA, and upsB/upsC) composition of the DBLα repertoires and the number of DBLα types per isolate for each of the six sentinel sites in Uganda. *Left hand y-axis*: For each sentinel site in Uganda the bar graph depicts each isolate’s DBLα repertoire major sequence group (upsA, and upsB/upsC) composition. The upsA proportion is represented by the use of lighter color tones in the upper portion of the column, whereas the upsB/upsC proportion is indicated by darker color tones in the lower portion. *Right hand y-axis:* Line graph depicting the number of DBLα types identified in each isolate. The line graphs have been ordered such that the minimum DBLα repertoire size (smallest number of DBLα types per isolate) is on the left and the maximum DBLα repertoire size (largest number of DBLα types per isolate) is on the right for each sentinel site.

For the 501 isolates analyzed for the six sentinel sites the size of DBLα repertoires ranged from the minimum DBLα type cut off of 20 (described above) to a maximum of 262 DBLα types per isolate (Table 1). The mean DBLα repertoire size was 101 DBLα types per isolate in all sentinel sites. In the low to moderate transmission sentinel sites (Kanungu, Jinja, Kyenjojo) the mean number of DBLα types in each isolate ranged from 68 to 98. In the high transmission sites (Arua, Tororo, Apac) the mean number of DBLα types in each isolate ranged from 113 to 121.We observed more than 60 DBLα types per isolate in 376 isolates (75.0%) consistent with the presence of multiple-clone infections in the majority of the population (Fig. 2).

When further considering the upsA and upsB/upsC composition for each DBLα repertoire, the proportions were similar within each sentinel site despite (i) the varying transmission intensities across Uganda and (ii) the number of DBLα types per isolate (i.e. DBLα repertoire size) (Fig. 2). The mean upsA and upsB/upsC DBLα type repertoire proportion for all sites was 0.16 upsA DBLα types (0.84 upsB/upsC). In each site the proportions ranged from 0.13 upsA DBLα types (0.87 upsB/upsC) in Kyenjojo to 0.18 upsA DBLα types (0.82 upsB/upsC) in Apac.

**Figure 3:**
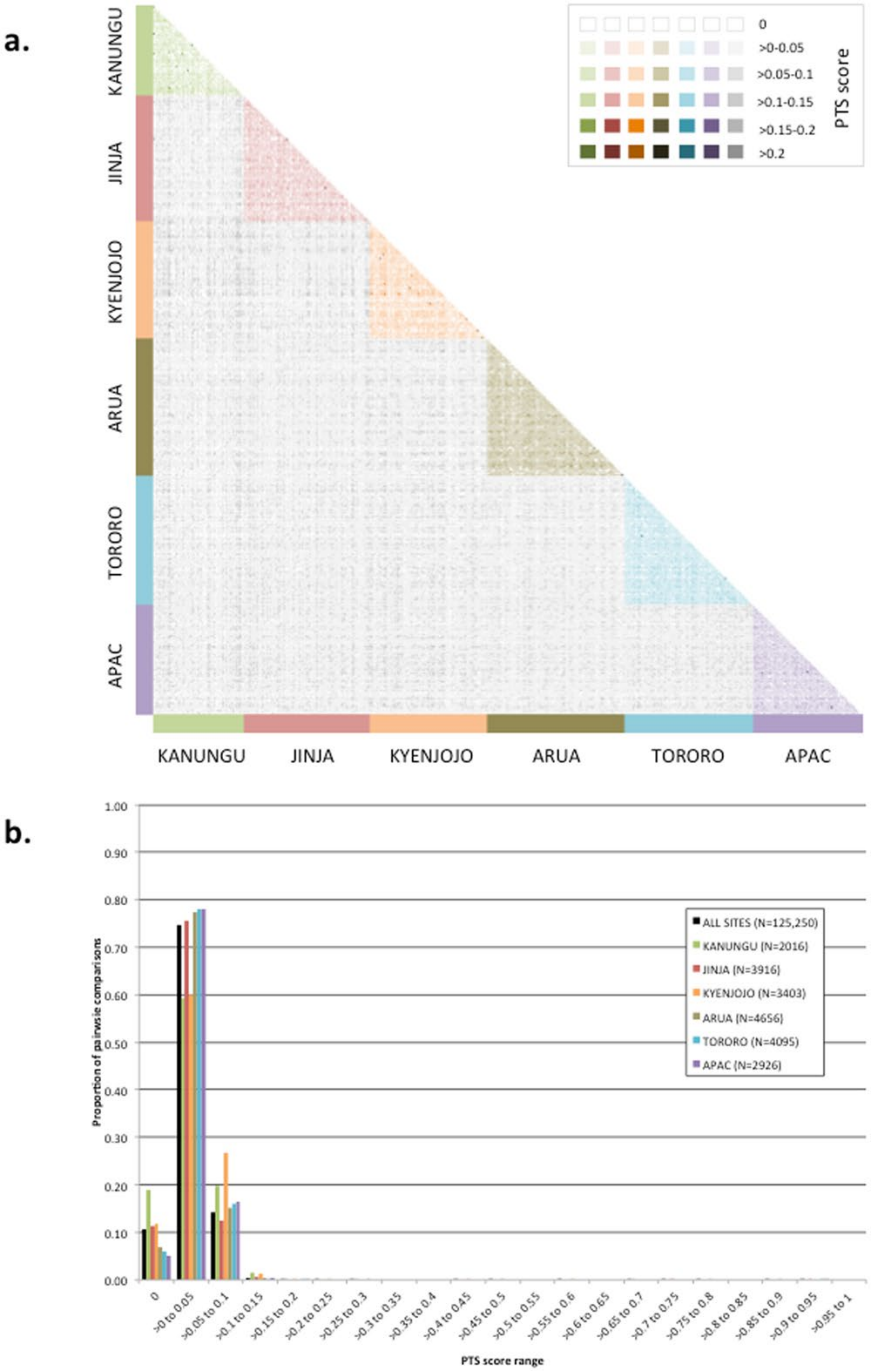
(**a**) Heat map representation of the PTS of DBLα types among isolates within and among sentinel sites in Uganda. The DBLα repertoire in one isolate was compared to the DBLα repertoire in all other isolates within a site and among all sites. Different shading colors (Green = Kanungu; Red = Jinja; Orange = Kyenjojo; Taupe = Arua; Blue = Tororo; Purple = Apac; Grey = sharing between sites) are used to indicate the PTS values by site and among all sites. The darker the color of the box, the greater the total number of shared DBLα types in the DBLα repertoires between two isolates: no shading represents a PTS score of zero (i.e., no sharing), the darkest signifies a PTS score >0.2 (indicates >20% sharing of DBLα types in the DBLα repertoires between two isolates) and gradation in-between represent PTS scores as indicated in the color key provided. (**b**) The frequency distribution of the proportion of pairwise comparisions within particular PTS score ranges within and among all sites. N refers to the total number of pairwise comparisons between isolates within each sentinel site and among all sites. The proportion estimates were calculated as follows: *# of pairwise comparisons within a specific PTS score range/total # pairwise comparisions in each site or among all sites*.

To determine whether epidemics of genomes with highly related DBLα repertoires were causing uncomplicated disease, we used pairwise type sharing (PTS) comparisons to examine the overlap of DBLα repertoires between isolates within and among the sentinel sites. Briefly, the DBLα repertoire in one isolate was compared to the DBLα repertoire in all other isolates within a site and among all sites. As mentioned above, 75.0% of isolates contained more than one parasite genome. Previous studies have shown that even when a parasite isolate contains more than one *P. falciparum* genome, PTS is a robust estimator of relatedness between isolates^12^. The presence of highly related genomes that share many DBLα types would be evidenced by high PTS scores between isolates. PTS among the 501 isolates resulted in 125,250 pairwise comparisons, of which 13,245 (10.6%) shared no DBLα types (Fig. 3). For 93,649 (74.8%) pairwise comparisons, the PTS score ranged from >0 to ≤0.05, indicating there was minimal sharing between all isolates (≤5% sharing) (Fig. 3). The median and mean PTS scores were 0.026 and 0.029 respectively among all sites (median PTS range across all sites = 0.025–0.033; mean PTS range across all sites = 0.028–0.037), with a maximum PTS score of 0.93 among all sites (maximum PTS range across all sites = 0.25–0.93). Only 17 (0.014%) pairwise comparisons had a PTS score >0.2 indicating that a strikingly limited number of isolates shared greater than 20% of their DBLα types. The overlap of DBLα repertoires within each sentinel site followed a similar pattern with an average of 81.4% of within site comparisons having a PTS score greater than 0 but less than or equal to 0.05, indicating that there was ≤5% sharing between DBLα repertoires in the majority of isolates within each sentinel site. This observed type sharing (~5%) is an upper limit since it is possible that type sharing can occur between different parasite genotypes within isolates with more than one parasite genome. Overall these results show that in this Ugandan parasite population uncomplicated malaria cases in children were caused by genetically distinct parasites with weakly overlapping DBLα repertoires (as defined by a PTS score ≤0.20) both on a local and national scale despite varying levels of transmission intensity across Uganda.

To investigate whether there were any selective sweeps of specific *var* DBLα types we examined repertoire overlap based on the upsA and upsB/upsC DBLα types separately, as upsA DBLα types are expected to be more conserved relative to upsB/upsC DBLα types^24^. It is important to note that as expected the repertoire sizes being compared were smaller for the upsA comparisons as the upsA DBLα types comprised, on average, only 16% of the repertoires. For the upsA DBLα types the median and mean PTS scores were 0.06 and 0.06 respectively among all sites (median PTS range across all sites = 0–0.07; mean PTS range across all sites = 0.05–0.07), with a maximum PTS score of 1 among all sites (maximum PTS range across all sites = 0.35–1) (Supplementary Figure S4). For the upsB/upsC DBLα types, the median and mean PTS scores were 0.02 and 0.03 respectively among all sites (median PTS range across all sites = 0.02–0.03; mean PTS range across all sites = 0.02–0.03), with a maximum PTS score of 0.93 among all sites (maximum PTS range across all sites = 0.23–0.93) (Supplementary Figure S4). These low PTS values show no evidence of epidemics of genomes with highly related DBLα repertoires or selective sweeps of specific *var* genes, not even among the more conserved upsA DBLα types.

### Geographic population structure of DBLα types

**Figure 4:**
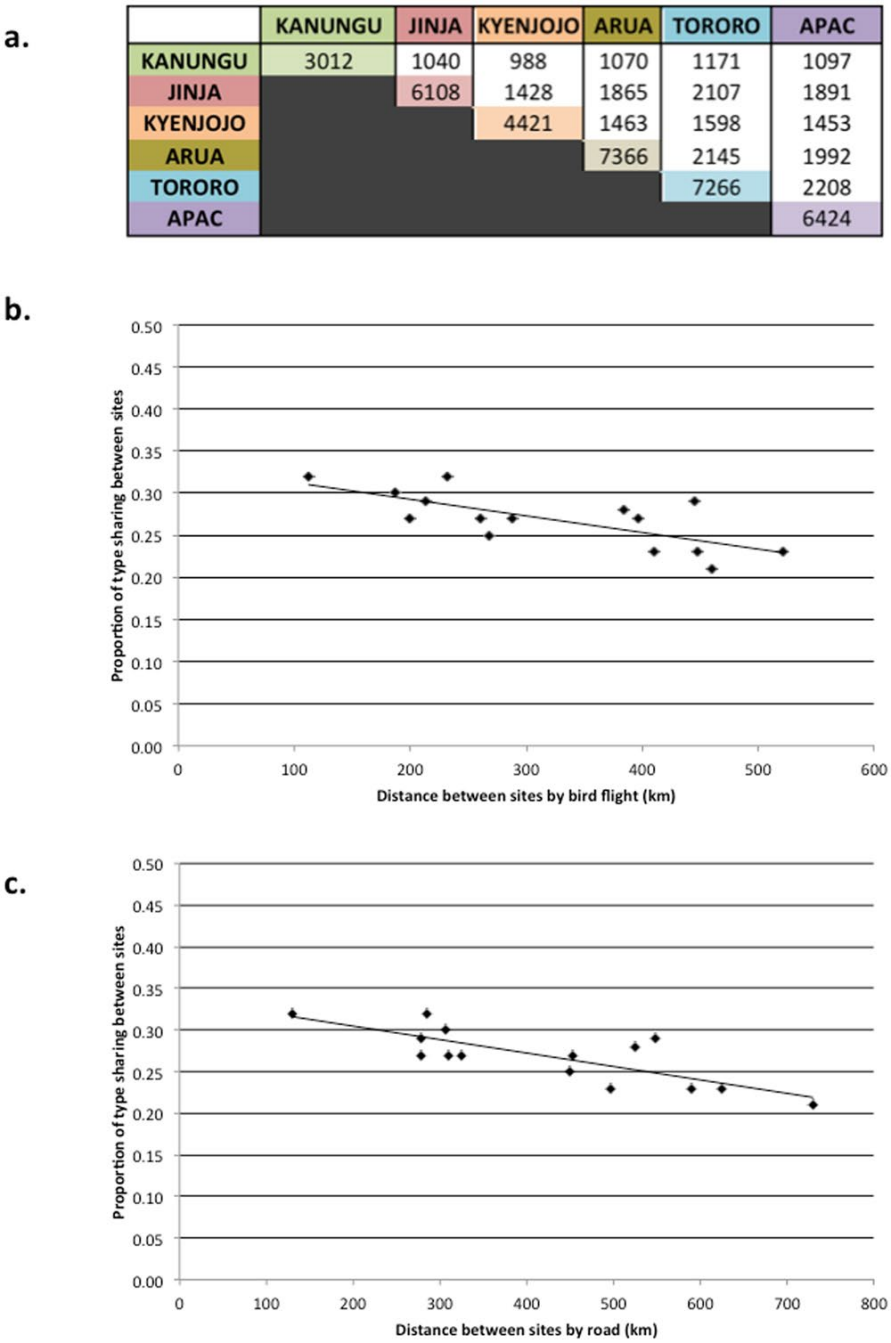
(**a**) Sharing of observed DBLα types (i.e., identical DBLα types) between the sentinel sites. (**b**) PTS (proportion) between each of the six Ugandan sentinel sites was compared to the distance between the sites using the “birds flight” and (**c**) road distance. The calculated Spearman’s rank correlation coefficients (ρ) were determined to be (**b**) −0.74 (*p* = 0.002) and (**c**) −0.72 (*p* = 0.003).

To assess the structuring of DBLα types on a geographic scale, we investigated the number of shared DBLα types between the sites. There was substantial sharing of DBLα types between sites regardless of the distance between them (Fig. 4a). The PTS scores of mean DBLα type sharing between two sites were also calculated and compared to the distance between the sites using “birds flight” and road distance. The distances between the sites ranged from 112.41 km (Jinja to Tororo) to 521.29 km (Tororo to Kanungu) by bird’s flight and from 130 km (Jinja to Tororo) to 729 km (Kanungu to Arua) by road distance (Supplementary Table S2). The calculated Spearman’s rank correlation coefficients (ρ) were determined to be −0.74 (*p* = 0.002) by bird’s flight and −0.72 (*p* = 0.003) by road (Fig. 4). The results from the distance analysis between sites using bird’s flight and road distance were statistically significant (*p* < 0.05), and indicate a negative correlation between distance and DBLα type sharing. Increasing the distance between sentinel sites decreases the sharing of DBLα types. Despite these trends, there was conservation of a large number of DBLα types, as defined by 96% sequence identity, across Uganda with 1,995 DBLα types seen in at least three sentinel sites and 277 DBLα types seen in all sites.

To take into account the patterns observed related to the upsA or upsB/upsC DBLα types, we investigated the number of shared DBLα types between sites independently for DBLα types classified as upsA and upsB/upsC. The same trend was observed for both upsA and upsB/upsC DBLα types with a negative correlation between distance and type sharing between sites, with sites farther apart sharing fewer DBLα types (Supplementary Figure S5). Interestingly, when the upsA and upsB/upsC DBLα types were analyzed independently, upsA DBLα type sharing between sites was significantly higher (range = 37–58% sharing, Supplementary Figure S5) compared to the upsB/upsC DBLα types (range = 18–29% sharing, Supplementary Figure S5) for all comparisons (*p* < 0.001). This is not altogether surprising, since upsA DBLα types have been reported to be more conserved, possibly due to balancing selection^24^.

## Discussion

*P. falciparum* causes a spectrum of malarial disease from asymptomatic to uncomplicated through to severe. Approximately 1–2% of children who become ill with malaria develop severe disease and only 0.25% of cases result in death^43,44^. Investigations of parasite virulence have associated the expression of semi-conserved clades of various parts of *var* gene sequences (rather than the presence of specific *var* genes in a genome *per se*) with defined disease outcomes, especially severe disease, in case control studies^26–34^. Unlike previous studies we have asked a different question: looking at the population genomics of *var* genes in cases of uncomplicated malaria, we set out to determine if there was any evidence of a selective sweep of specific *var* genes or epidemic structure related to the incidence of uncomplicated disease in children.

We investigated the diversity and population structure of *var* gene sequences encoding the *Pf* EMP1 DBLα domain of *P. falciparum* causing uncomplicated malaria. This was examined in six sites across Uganda with varying malaria transmission intensities, from low (i.e., low recombination rates) to high (i.e., higher recombination rates). Surprisingly, even in low transmission sites we found no evidence of a selective sweep of any specific *var* DBLα types in the parasites causing uncomplicated malaria, even after stratifying by both the upsA and upsB/upsC DBLα types. In fact, a key genetic feature of these parasites was that they all showed minimal overlap with respect to their DBLα repertoires suggesting that there is selection for *var* diversity and heterogeneity at the DBLα repertoire level in this population. Indeed, there was no particular common genetic signature indicative of clonal propagation or epidemic expansion of related *P. falciparum* parasites causing uncomplicated malaria as has been reported in Senegal^45^ where, in comparison to our study sites, malaria control has been intense.

Our analysis of 21,134 unique DBLα types from 501 uncomplicated cases revealed extensive diversity of the DBLα domain of *var* genes, consistent with Chao estimates of DBLα diversity from previous limited sampling that we completed in African sites with high transmission^13^. Paradoxically, we found high diversity at the DBLα type level yet conservation of individual DBLα types with 40.9% of the DBLα types identified being conserved in Uganda (i.e., seen more than once when all the six surveyed populations were combined). These striking results would not be predicted by the extremely high rates of mitotic recombination that have been reported from *in vitro* experiments with culture adapted *P. falciparum* lines^25^. Based on the conservation of DBLα types between isolates and across sites, we propose that there are forces structuring the parasite population to maintain these DBLα types in the transmission system. This would be consistent with a mechanism such as balancing selection^24^.

Although we observed extensive diversity at the DBLα repertoire level, the proportional representation of upsA and upsB/upsC DBLα types within each repertoire was maintained as expected^24^. This is likely due to their chromosomal orientation and reduced levels of recombination with other *var* gene groups to maintain domain architecture^46,47^. Interestingly, when looking at the frequency of the DBLα types across all sites, the upsA were ~9x more likely to be seen in 10 or more isolates when compared to the upsB/upsC. This significant pattern was only observed in the high transmission sites (EIR >300) where exposure to malaria is intense. Thus, the assumed increased rates of meiotic recombination in high transmission sites have led to the dispersal of the same upsA DBLα types into a greater proportion of *P. falciparum* repertoires.

Every DBLα repertoire was unique in the 501 uncomplicated malaria cases with minimal overlap of the DBLα repertoires within, between and across all sites. The observed parasite population structure further supports the immune selection model described by Artzy-Randrup *et al*.^23^. They explored the population structuring of *var* genes by simulating the dynamics of all possible *var* gene combinations given a large and diverse pool of *Pf* EMP1 variants as well as patterns of immunity in the host population. They showed that despite high levels of outcrossing (i.e., high meiotic recombination rates) there is a defined parasite population structure that exhibits “strain” structuring^23^. This is consistent with our observations as the *P. falciparum* parasites causing uncomplicated malaria have distinct, minimally overlapping DBLα repertoires despite varying transmission intensities across Uganda. Furthermore we observed the same pattern locally (i.e., within a site) and nationally (i.e., across all sites) as predicted by the immune selection model where the same *var* repertoire structuring would be expected despite different host population sizes^23^. This is also the same pattern of “strain” structure that we observed in isolates from asymptomatic children in Bakoumba where a clear *var* population structure with limited overlap in the DBLα repertoires was described^15^. This structure would enhance the parasites’ ability to evade the host immune response under conditions of high transmission and high recombination rates.

A trade-off between maintaining parasite fitness in the host while still achieving successful transmission between hosts (i.e., immune evasion), has been described by Buckee and Recker^24^. They proposed an evolutionary model where parasite phenotypic plasticity could explain population structuring such that there is a trade-off of evolutionary strategies: conservation of *var* gene domain architecture (i.e., sequence length) serves to optimize parasite fitness and diversity at the *var* repertoire level maximizes immune evasion. Sequencing results from our study show for the first time both conservation of individual DBLα types (at 96% sequence identity) and yet high repertoire diversity in uncomplicated *P. falciparum* cases. The evolutionary forces maintaining this pattern of diversity need to be explored further with models that consider the large number of DBLα variants we have seen in nature. There is no question that the need to prolong infection so as to enable transmission requires evasion of the host immune system, which provides a strong selection force to diversify *var* genes as well as repertoires within a host.

Many studies have shown that individuals with uncomplicated malaria exhibit a broad range of antibody responses and harbor parasites that express diverse *Pf* EMP1 variants^27,28,31,46,48,49^. Although we define sequences of the same DBLα type as sharing 96% sequence identity, epitopes can be shared between sequences with a lower identity (e.g. the DBLβ3 domains of DC4 share 80% sequence identity but are antigenically cross-reactive^50^). Nonetheless, the prevalence of the host variant-specific anti-*Pf* EMP1 response will be dependent on the extent of *Pf* EMP1 variant diversity in the parasite population. Hence, acquisition of immunity to the less diverse upsA DBLα types would be expected to occur faster than the ~12x more diverse upsB/upsC DBLα types. Serological network studies by Buckee *et al*. (2009) propose that immune selection occurs at different levels within the upsA or upsB/upsC groups of the same *var* multigene family^51^ and this could influence the acquisition of immunity to specific *var* gene groups.

When considering geographic structuring of conserved DBLα types there was significantly higher sharing of upsA DBLα types (range 37–58%) between sites compared to the upsB/upsC DBLα types (range 18–29%) over the large geographic distances sampled in Uganda (~100 km to ~600 km). Paradoxically given this significant conservation of the upsA DBLα types across sites, we still report minimal PTS overlap of DBLα repertoires between isolates. This pattern of PTS was consistent no matter how we partitioned the repertoires per isolate: (i) the entire DBLα repertoire, (ii) only the upsB/upsC DBLα types, and perhaps most interestingly, (iii) even when exclusively examining the upsA DBLα types. This highlights the fact that there was no detectable evidence of linkage between the DBLα types at the isolate repertoire level.

We observed a paradoxical pattern of high diversity and minimal overlap at the parasite *var* DBLα repertoire level within a host, yet conservation of 40.9% of DBLα types. This overall pattern was observed repeatedly by our sampling at multiple sites in Uganda and was the same regardless of geographic distance or malaria transmission intensity. Whether demographic forces or immune selection are maintaining this pattern remains to be answered. Importantly, the low PTS among *var* DBLα repertoires show that any parasite with a diverse repertoire of *var* genes, rather than an epidemic of parasites with related *var* repertoires, can cause uncomplicated disease.

## Methods

### Ethical Statement

The study was reviewed and approved by the Uganda National Council of Science and Technology and by the institutional review boards/human ethics committees of Makerere University (Kampala, Uganda), University of California (San Francisco, US), New York University School of Medicine (New York, US), and The University of Melbourne (Melbourne, AU). Written informed consent was obtained in the local language from the parent/guardian(s) for the children enrolled in this study and for the future use of the biological specimens. Parent/guardian(s) of the children were asked to volunteer and were not coerced. The researchers in the study had no professional relationship with the individuals recruited for this study. Biological specimens collected consisted of dried blood spots on filter paper collected by trained personnel at the hospital. The consent process was consistent with the ethical expectations at the time of enrollment and the ethics committees approved these procedures.

### Study sites and population

The study was performed in six independent sentinel health centers established by the Uganda Ministry of Health. Malaria endemicity is widely variable across Uganda and the six sentinel study sites were chosen to represent the geographic diversity of malaria transmission intensity: based on the annual entomological inoculation rates (EIR) Kanungu, Jinja and Kyenjojo experience a low to moderate transmission (EIR = 6, 6, 7, respectively) whereas the study sites Arua, Tororo and Apac experience relatively higher transmission intensities (EIR = 397, 563, 1586, respectively). A cross-sectional survey was used to consecutively enroll 1000 outpatients per sentinel site who had been referred to the laboratory for a diagnostic malaria blood smear in accordance with the standard of care for fever case management between May 2006 and February 2007. Details on the study population and data collection procedures have been published elsewhere^52^. Briefly, after obtaining informed consent from all individuals and/or their parents/guardians, the participants’ age and sex were recorded and a finger-prick blood isolate was obtained for a thick film blood smear, a rapid diagnostic test (RDT) and a dried blood spot (DBS) for molecular testing. From the 1000 outpatients who were enrolled at each of the six sentinel sites across Uganda (N = 6000), isolates from 100 children between 6 months and 5 years from each of the six sentinel sites (N = 600) with a microscopy confirmed *P. falciparum* infection were included in this analysis. For the purposes of this study all children who tested positive for *P. falciparum* by microscopy, were febrile (≥37.5 °C), and showed no additional symptoms to indicate severe disease when enrolled were defined as having an “uncomplicated *P. falciparum* infection”.

### DNA extraction

Genomic DNA from each DBS isolate was extracted using the QIAamp DNA Mini Kit (Qiagen, Valencia, CA) according to the manufacturer’s instructions.

### *Var* DBLα PCR and sequencing

The *P. falciparum var* genes from genomic DNA were amplified using the DBLα domain as previously described with modifications^12,13^. From each isolate of genomic DNA, a ~550–700 bp region of the DBLα domain was amplified using a degenerate primer set (F*: 5*′*-CMTGYGCDCCRTWYMGAMG, R: 5*′*-TCKGCCCATTCYTCRAACCA*) designed against the semi-conserved blocks B and H of DBLα^8^. Each of the DBLα primers were designed by adding a GS FLX Titanium primer sequence 10 bp multiplex identifiers (MID) published by Roche^53^. These MID primers were used to ‘barcode’ and distinguish the DBLα sequences amplified from a unique isolate once all isolates were pooled and sequenced together^54^. The PCR conditions for the DBLα amplification were as follows: 2 μl of isolate genomic DNA, dNTPs at a final concentration of 0.07 mM, each primer (forward and reverse with same MID combination) at a final concentration of 0.375 μM, MgCl_2_ at a final concentration of 2 mM, 1x reaction buffer, and 3 units GoTaq Flexi polymerase (Promega) in a 40 μL total reaction volume. PCR cycling was carried out on an Eppendorf thermal cycler and involved an initial denaturation step of 95 °C for 2 min, 30 cycles of 95 °C × 40 sec, 49 °C × 90 sec, and 65 °C × 90 sec, followed by a final extension step of 65 °C for 10 min. Finally the isolate amplicons were pooled and sequenced using next generation 454 sequencing (Roche) performed at NYU School of Medicine at the Center for Health Informatics and Bioinformatics and the Memorial Sloan-Kettering Cancer Center Genomics Core Laboratory. The 454 sequencing provides average read lengths of 400 bp, therefore lending itself to the assembly of the individual *var* DBLα amplicons of 550–700 bp lengths using the forward and reverse sequence reads from each direction.

### DBLα sequence analysis

A custom pipeline was developed to de-multiplex, de-noise and remove PCR and sequencing artefacts from the DBLα domain reads. The first part of the pipeline is available as the Multipass web server: http://www.cbs.dtu.dk/services/MultiPass-1.0, and the following cleaning steps described below are implemented in a python script available here: https://github.com/454data/postprocess. The sff-files obtained from each region on the 454-plate were divided into smaller isolate specific sff-files by identification of reads with exact matching MID sequences in both ends using BioPython v1.57. Ambiguous primer sites were then identified (exact match) and trimmed off the flowgrams, reverse reads were reverse complemented, and a dat-file (AmpliconNoise format) with the resulting flowgrams was created for each isolate, using BioPython v1.^55^. By combining the forward and reverse reads this method takes advantage of bi-directional amplicon sequencing, since the forward reads will have highest quality in the 5′-end of the target sequence, and the reverse reads will improve the 3′-end quality. Flowgram clustering was performed using PyroDist, FCluster and PyroNoiseM from the AmpliconNoise package v1.25^56^. The flowgram clusters produced by AmpliconNoise were base called using Multipass to obtain the most likely *var* DBLα sequences given the flowgrams and a high open reading frame likelihood, as described in ref.^57^. The nucleotide sequences generated by Multipass were clustered by 96% identity using Usearch v5.2.32^58^ with seeds (cluster member with support from highest number of reads after de-replication) as output. Chimeras were removed using Uchime implemented in Usearch v5.2.32^58,59^, first in de-novo mode where chimera detection is based on read abundance, all parents are expected to be present in the sequence set, and candidate parents must be at least 2x more abundant than the chimera candidate sequence; subsequently in database mode, where sequences are searched against self and chimeras are found irrespective of the abundance of the parents. To increase overall quality of the sequences remaining at this point, a minimal coverage threshold of three reads per sequence type was applied to remove the least supported sequences. Next, we screened for and removed non-target amplified human sequences by local alignment search against the BLAST human genomic databases (http://ftp.ncbi.nlm.nih.gov/blast/db/) using the blastn feature of BLAST+ 2.2.25 (NCBI), with expectation value criteria of 1e-50. Sequences were also searched using a DBLβ HMM with HMMer v3.1 (hmmer.org). After the human and non-target *P. falciparum* check, a small number of sequences remained that had no similarity to a DBLα-tag HMM and these were removed. The pipeline was validated and optimized on experimental sequence data generated on the laboratory clones (3D7, Dd2, and HB3) for which published genome sequence is available. More than 90% of the sequences obtained from the control samples had no errors when compared to the known reference, and the deviating sequences had maximally 5 errors. To subsequently determine DBLα types shared between isolates, the cleaned DBLα reads were clustered using a pipeline based on the USEARCH software suite version 8.1.1831^58^. Initially duplicate reads were removed and the remaining reads were sorted by how many duplicates were present using the derep_prefix command. The remaining reads were then clustered at 96% pairwise identity using the usearch cluster_fast command. Finally, the original unfiltered reads were aligned back to the centroids of the clusters and an OTU table was generated using the usearch_global command before a binary version of the table was generated. The code for the pipeline is available on GitHub at https://github.com/UniMelb-Day-Lab/clusterDBLalpha.

### Cumulative diversity curves

The cumulative diversity curves, analogous to species accumulation, were generated using EstimateS v9.1^60^ to estimate *var* DBLα richness by sampling all DBLα types from each sentinel site and among all sites without replacement. All sampling depths between one and the number of DBLα types obtained from the study site were repeated 100-fold, whereupon the mean number of *var* DBLα types obtained for each sampling depth was calculated. The cumulative diversity curve was plotted using R v3.13^61^ to plot the number of *var* DBLα types as a function of the number of *var* DBLα sequences sampled.

### Frequency plots

Frequency plots were tabulated using the data generated and plotted using Microsoft Excel.

### Richness estimates

Using EstimateS v9.1^60^ the diversity of DBLα types within and among all sites in Uganda were calculated by estimating the total number of DBLα types and the proportion of DBLα types shared between isolates. For each sentinel site two different statistical estimates of richness were used, non-parametric statistical Chao2 and incidence-based coverage estimator (ICE). Chao2 calculations were used to estimate the total number of DBLα types in a particular sentinel site based on singletons and doubletons^62,63^. ICE statistics were used to estimate the total number of DBLα types in a study site based on all the observed types by dividing them into two groups: rare and abundant types^64^. Jacknife2 calculations were used to estimate the total number of DBLα types in a particular sentinel site by resampling the unique and duplicate types, thereby reducing bias^41^.

### Pairwise type sharing

For each sentinel site, pairwise type sharing (PTS) statistics were calculated to quantify the relatedness between the DBLα repertoires identified from two isolates. This methodology has been published elsewhere^12,13^ and is a useful statistic to analyze diversity and determine the number of DBLα types shared between isolates. Briefly, PTS is calculated as follows:

1 $$PT{S}_{isolate}=\frac{2\times ({\rm{number}}\,{\rm{of}}\,{\rm{shared}}\,\mathrm{DBL}{\rm{\alpha }}\,{\rm{types}}\,{\rm{in}}\,{\rm{isolates}}\,{\rm{A}}\,{\rm{and}}\,{\rm{B}})}{{\rm{number}}\,{\rm{of}}\,\mathrm{DBL}{\rm{\alpha }}\,{\rm{types}}\,{\rm{in}}\,{\rm{isolate}}\,{\rm{A}}+{\rm{number}}\,{\rm{of}}\,\mathrm{DBL}{\rm{\alpha }}\,{\rm{types}}\,{\rm{in}}\,{\rm{isolate}}\,{\rm{B}}}$$

Thus, a PTS score is the ratio of the number of shared DBLα types between two isolates and the sum of DBLα types in both isolates. The ratio ranges between 0 and 1, where a PTS score of 0 signifies no DBLα repertoire similarity and 1 signifies identical DBLα repertoires.

### Distance-based pairwise type sharing

PTS statistics were calculated **t**o estimate the mean DBLα type sharing between two sentinel sites. PTS scores between sites were calculated as follows:

2 $$PT{S}_{site}=\frac{2\times ({\rm{number}}\,{\rm{of}}\,{\rm{shared}}\,\mathrm{DBL}{\rm{\alpha }}\,{\rm{types}}\,{\rm{in}}\,\mathrm{sites}\,1\,{\rm{and}}\,2)\,}{{\rm{number}}\,{\rm{of}}\,\mathrm{DBL}{\rm{\alpha }}\,{\rm{types}}\,{\rm{in}}\,\mathrm{site}\,1+{\rm{number}}\,{\rm{of}}\,\mathrm{DBL}{\rm{\alpha }}\,{\rm{types}}\,{\rm{in}}\,{\rm{site}}\,2}$$

Thus, a PTS score is the ratio of the number of shared DBLα types between two sites and the sum of DBLα types in both sites. A comparison was performed to evaluate the effect of distance (“birds flight” distance and transit/road distance) on DBLα type sharing between sentinel sites. “Birds flight” distance between the sentinel sites was calculated using Google Maps (https://www.google.com/maps/preview) and transit/road distance was calculated using the Via Michelin Maps and Routes application (http://www.viamichelin.com). Spearman’s rank correlation coefficient was calculated to evaluate the trend and was performed using IBM SPSS (Version 22) software.

### Classification of *var* gene A/non-A groups

Reads were translated into all six reading frames. Protein hidden Markov models of 150 VAR domains of Rask *et al*.^11^ were aligned to the translated sequences using HMMER v3.1b1 with an e-value of 0.01. Both the bias composition correction and composition filter were turned off as suggested in Rask *et al*. (–nonull2 —tobias)^11^. The most significant match to any of a reads six translated frames was then taken as the domain assignment for that read. Reads were further classified into upsA if their most significant match was with a DBLα1 domain and upsB/upsC (i.e., non-A) if that matched with either DBLα0 or DBLα2 domains. The domain models did not allow for an accurate distinction between *var* groups B and C. Cross validation was used to ascertain the success of this approach with the classification accuracy of upsA and upsB/upsC combined found to be 96.2% and 99.8%, respectively (see Supplementary Text 1).

### Statistical analysis

Statistical analyses were carried out using IBM SPSS Statistics (Version 22) software and the open source EpiInfo v7 (https://www.cdc.gov/epiinfo/index.html) software. A test was deemed to be statistically significant if the *p-value* was less than 0.05. For all analyses the sentinel sites (Kanungu, Jinja, Kyenjojo, Arua, Tororo, and Apac) were categorized as defined in the study design. Chi-squared test (*χ* ^2^) was used for univariate analyses of discrete variables to compare proportions.

### Data Availability Statement

The nucleotide sequences reported in this paper have been deposited in the NCBI database (Project no. PRJNA385208, Accession no. SAMN06833355-SAMN06833855).

## Electronic supplementary material

Supplementary Information
