# Supplementary material for: Population genomics of virulence genes of Plasmodium falciparum in clinical isolates from Uganda
Source: Sci Rep. 2017 Sep 18;7:11810. doi: 10.1038/s41598-017-11814-9 (PMC5603532; doi:10.1038/s41598-017-11814-9)
Supplement: Supplementary file 1 — Supplementary Information [file 41598_2017_11814_MOESM1_ESM.pdf]

Population genomics of virulence genes of *Plasmodium falciparum* in clinical isolates from Uganda.

Shazia Ruybal-Pesántez*[1,2], Kathryn E. Tiedje*[1,2], Gerry Tonkin-Hill[3], Thomas Rask[1,2], Moses R. Kamya[4], Bryan Greenhouse[5], Grant Dorsey[5], Michael F. Duffy [1,] and Karen P. Day[1,2]**

[1] *School of BioSciences, Bio21 Institute/University of Melbourne, Melbourne, AU*
[2] *Department of Microbiology, New York University, New York, USA*
[3] *Walter and Eliza Hall Institute, Melbourne, AU*
[4] *School of Medicine, Makerere University College of Health Sciences, Kampala, Uganda*
[5] *Department of Medicine, University of California, San Francisco, USA*

*** Co-first authors.**
****Corresponding author.**

SUPPLEMENTARY INFORMATION

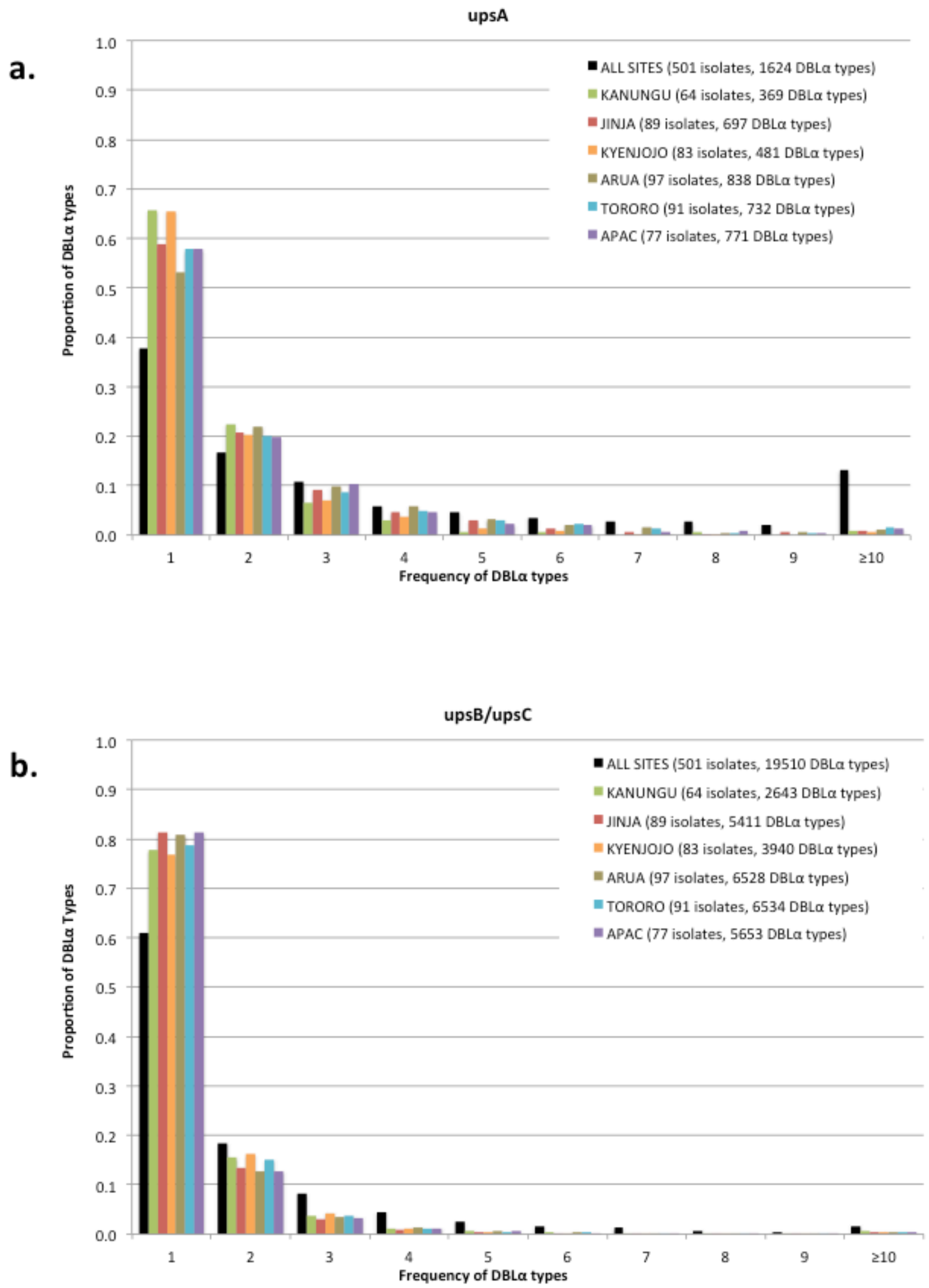

Figure S1. Proportion of **a)** upsA DBLα types and **b)** upsB/upsC DBLα types appearing 1 to 10 or more times within and among all sentinel sites.

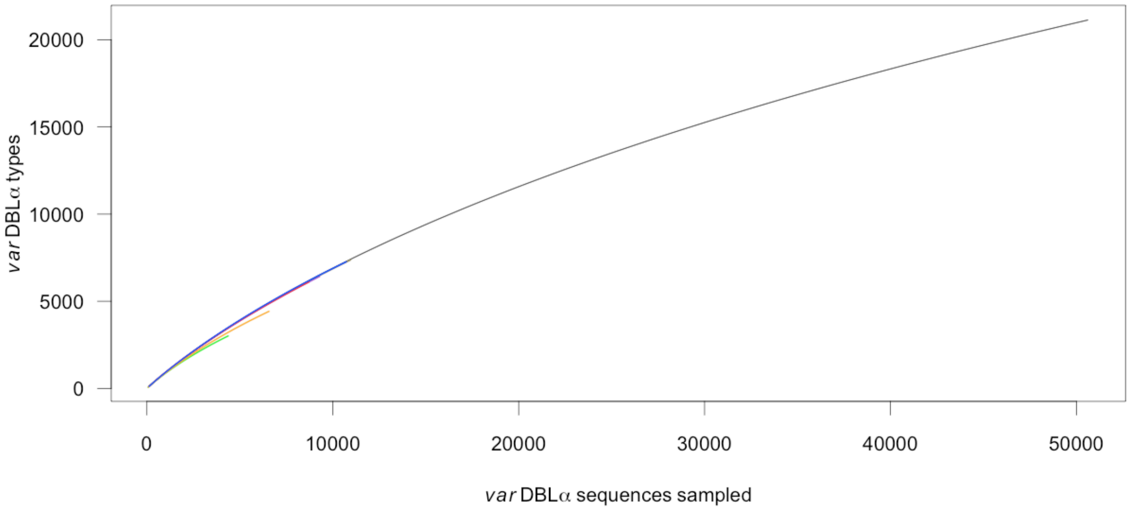

Figure S2. Cumulative diversity curves within and among all six sentinel study sites in Uganda; plotting the number of *var* DBLα sequences sampled against the number of observed *var* DBLα types. A DBLα type was defined by a 96% sequence identity threshold and corresponds to a unique DBLα sequence. The color of the lines indicate as follows: Black = All Sites, Green=Kanungu; Red=Jinja; Orange=Kyenjojo; Taupe=Arua; Blue=Tororo; Purple=Apac.

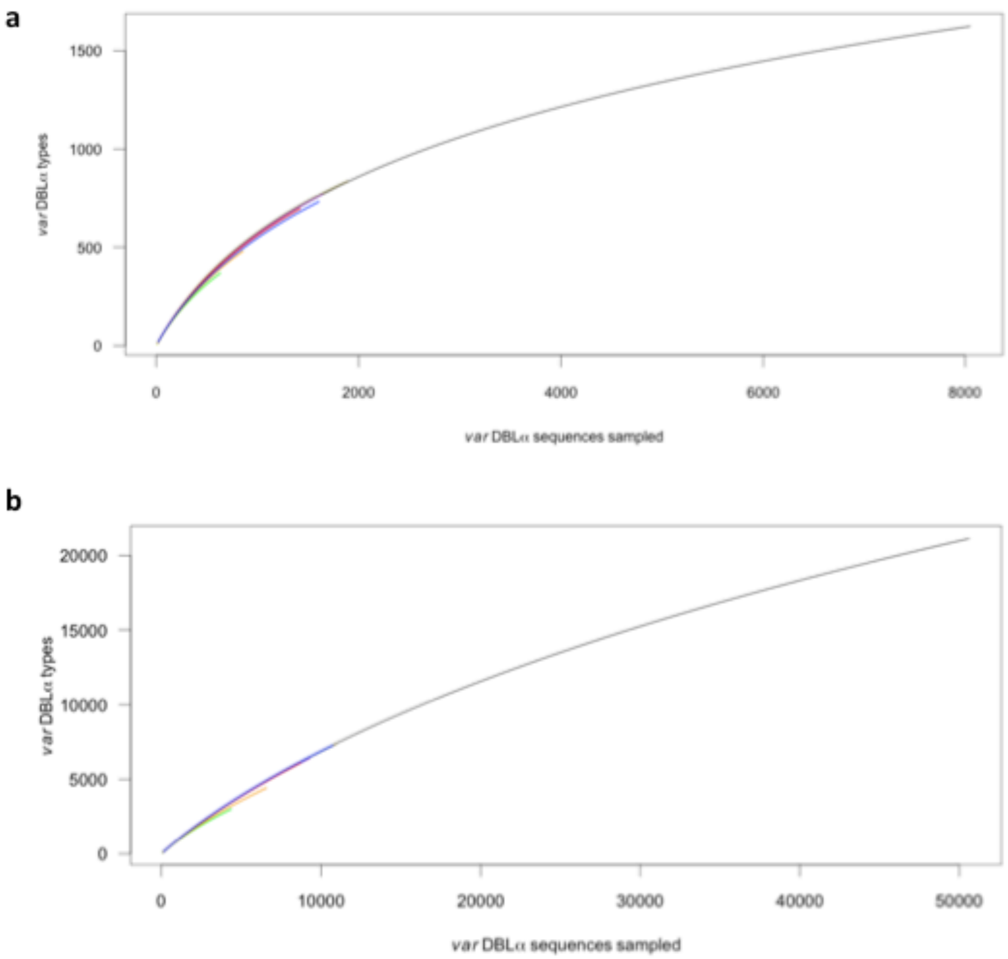

Figure S3. Cumulative diversity curves within and among all six sentinel study sites in Uganda; plotting the number of *var* DBLα sequences sampled against the number of observed *var* DBLα types for **A.** upsA DBLα types and **B.** upsB/upsC DBLα types. The color of the lines indicate as follows: Black = All Sites, Green=Kanungu; Red=Jinja; Orange=Kyenjojo; Taupe=Arua; Blue=Tororo; Purple=Apac.

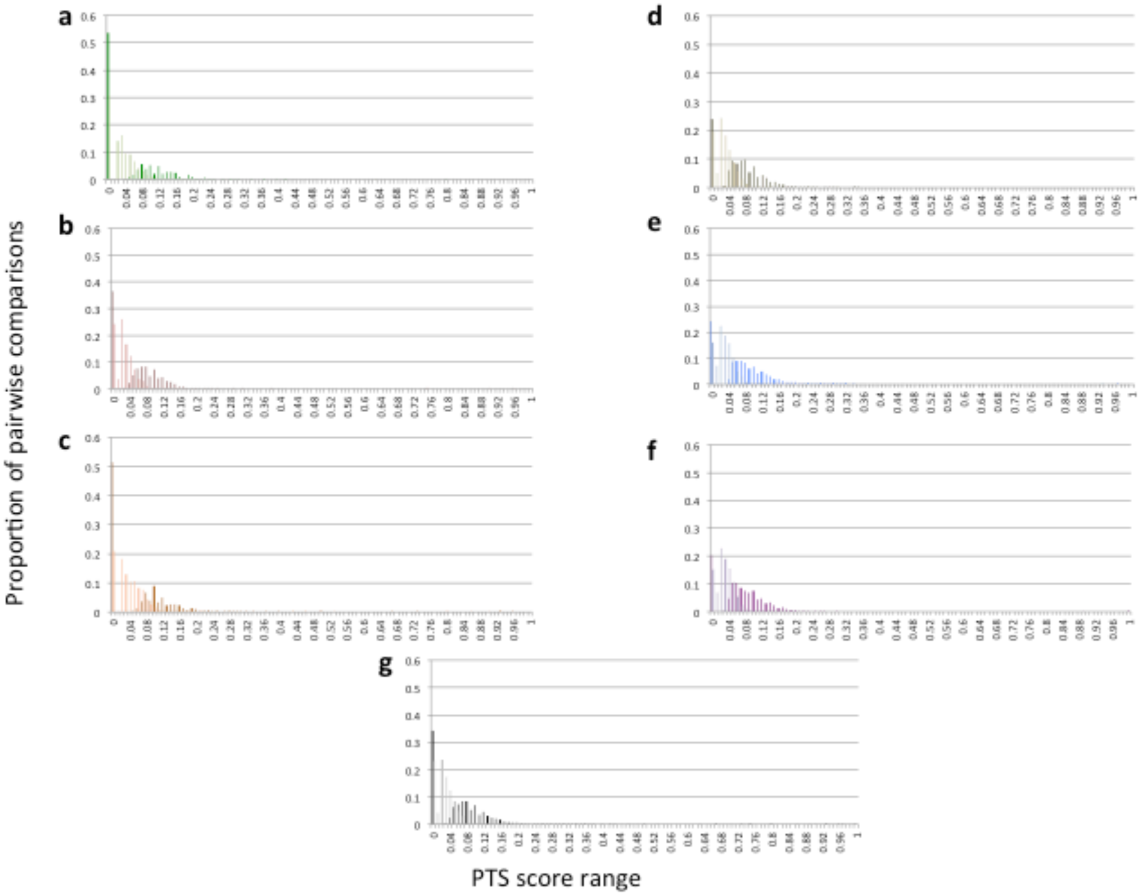

Figure S4. The frequency distribution of the proportion of pairwise comparisions within particular PTS score ranges within **A.** Kanungu **B.** Jinja **C.** Kyenjojo **D.** Arua **E.** Tororo **F.** Apac and **G.** among all sites. The upsA proportions are represented by the use of lighter color tones, whereas the upsB/upsC proportions are indicated by darker color tones.

a.

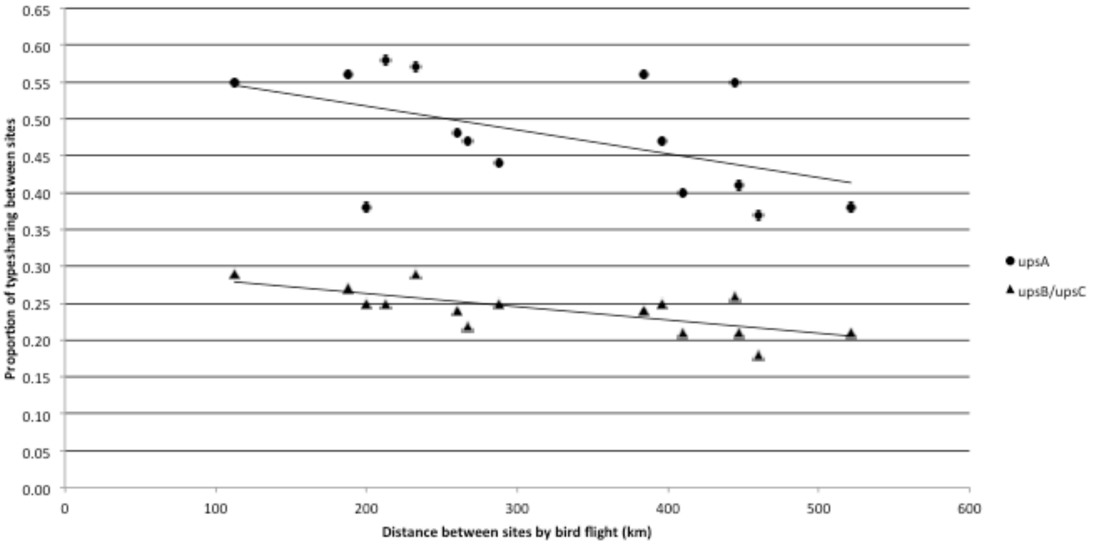

b.

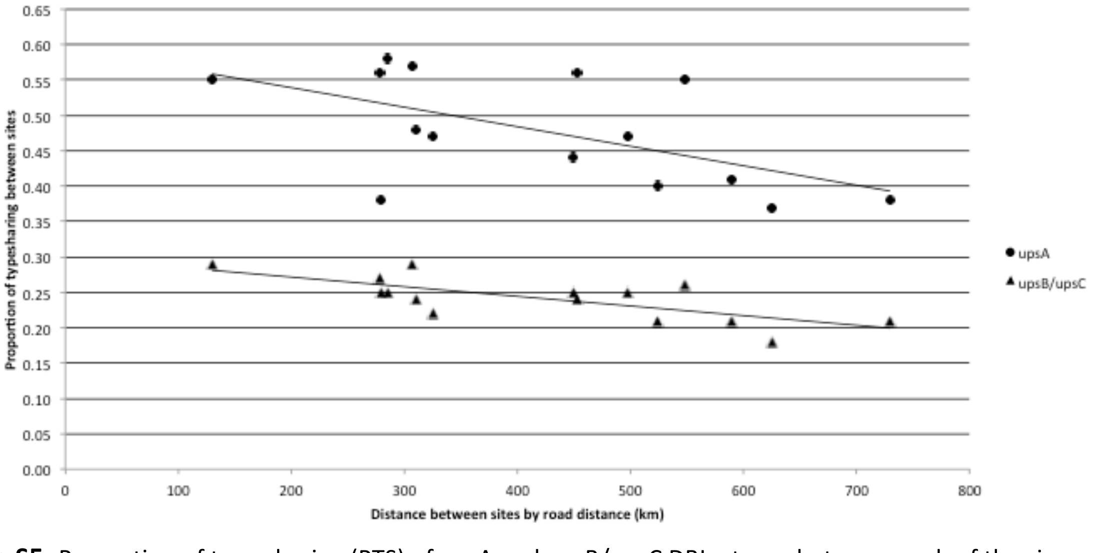

Figure S5. Proportion of type sharing (PTS) of upsA and upsB/upsC DBLα types between each of the six Ugandan sentinel sites was compared to the distance between the sites using the **A.** bird flight and **B.** road distance.

Table S1. *Var* DBLα richness estimates by sentinel study site**.**

**Study Sites**	**Chao2 richness estimate (95% CI)**	**Proportion of total DBLα types sampled** [a]	**ICE richness estimate**	**Jackknife2 richness estimate**
Kanungu	8,278 (7,668-8,968)	0.364	9,536	7,031
Jinja	19,364 (18,236-20,598)	0.315	22,459	14,742
Kyenjojo	11,914 (11,190-12,715)	0.371	13,549	10,270
Arua	23,366 (22,108-24,731)	0.315	27,045	17,653
Tororo	20,879 (19,828-22,017)	0.348	24,709	17,125
Apac	20,801 (19,592-22,120)	0.309	23,726	15,474
**All Sites**	41,345 (40,376-42,363)	0.511	44,092	42,207

[a] Calculated by the following formula: *proportion DBLα types sampled=* total *no. DBLα types/Chao2 richness estimate*.

Table S2. Distances between sentinel study sites.

**Site 1**	**Site 2**	**Bird's flight distance (km)**	**Road distance (km)**
Jinja	Tororo	112.41	130
Jinja	Apac	187.67	278
Kyenjojo	Kanungu	199.54	279
Apac	Arua	212.98	285
Tororo	Apac	232.24	307
Kyenjojo	Apac	260.21	310
Kyenjojo	Arua	267.56	325
Jinja	Kyenjojo	288.1	449
Jinja	Arua	383.48	453
Tororo	Kyenjojo	395.82	497
Jinja	Kanungu	409.55	524
Tororo	Arua	444.78	548
Kanungu	Apac	446.93	589
Kanungu	Arua	460	625

Supplementary Text 1 - DBLa HMMER domain identification

Libraries

```
library(data.table)
library(ggplot2)
library(stringr)
library(dplyr)
```

Here we show that the hidden Markov model approach using HMMER accurately classifies the DBLa reads into either UPS type A or a combined category of UPS types B and C.

First we determine the average read length in order to simulate reads to align.

```
from mungo.fasta import FastaReader
import numpy as np

l=[]
for h,s in FastaReader("./uganda_454_reads.fasta"):
  l.append(len(s.replace("-","")))

np.mean(l)/3
```

The mean read length was found to be approximately 370 base pairs giving an equivalent protein length of approximately 123aa.

We made use of cross validation to determine the accuracy of the HMMER hidden Markov models in classifying DBLa reads. Simulated translated protein reads were generated from each domain in the HMMER model database (Rask et al, 2010). A new HMMER model database was then created excluding this domain. Finally, the simulated protein reads were aligned to the new HMMER model database.

In the manuscript we translate the DNA reads into all 6 frames and take the best hit. As it was deemed very unlikely that the incorrect reading frame would give a higher classification score we did not simulate from DNA.

```
mkdir temp_output
```

The python code below implements the cross validation procedure.

```
from mungo.fasta import FastaReader
import glob
import random
import os, sys
from subprocess import check_call

OUTDIR = "temp_output/"
READ_LENGTH=123


def sampleReads(string, length):
  #Deal with short domains
  while(len(string)<length):
    length = length-50
```

```
  nStart = random.choice(range(len(string)-length))
  return string[nStart:(nStart+length)]

def makeHMMER_withoutDomain(MSA, domain):
  domain_class = os.path.splitext(os.path.basename(MSA))[0]
  new_msa_file = OUTDIR + domain_class + "MSA.fasta"
  with open(new_msa_file, 'w') as outfile:
    for h,s in FastaReader(MSA):
      if h==domain:
        print "REMOVING"
        continue
      outfile.write(">"+h+"\n"+s+"\n")
  check_call("hmmbuild " + OUTDIR + domain_class + ".hmm " + new_msa_file
    , shell=True)

for msa in glob.glob("./MSA_alignments/*.fasta"):
  domain_class = os.path.splitext(os.path.basename(msa))[0]
  cmd = "hmmbuild " + OUTDIR + domain_class + ".hmm " + msa
  print cmd
  check_call(cmd, shell=True)

for msa in glob.glob("./MSA_alignments/*.fasta"):
  if not "DBLa" in msa: continue #only simulate DBLa reads
  for h,s in FastaReader(msa):
    dom_filename = h
    dom_filename = dom_filename.replace(" ", "_")
    dom_filename = dom_filename.replace("/", "")
    seq = s.replace("-","")
    if len(seq)<10:
      print msa,h,s
    #Sample 10 artificial reads from domain
    sample_reads = OUTDIR + dom_filename + "_sampleReads.fasta"
    with open(sample_reads, 'w') as outfile:
      for i in range(10):
        outfile.write(">read"+str(i)+"_"+dom_filename+"\n"+ sampleReads(seq, READ_LENGTH) + "\n")
    #Make new hmmer file excluding domain h
    makeHMMER_withoutDomain(msa, h)
    #concatenate hmmer files for search
    cmd = "cat " + OUTDIR + "*.hmm > " + OUTDIR + "combined.ALLhmm"
    print cmd
    check_call(cmd , shell=True)
    #Now search artificial reads against hmmer models
    cmd = ("hmmsearch --tblout " + OUTDIR + dom_filename + "_hmmSearch.txt"
            + " -E 1e-8 --nonull2 --nobias "
            + OUTDIR + "combined.ALLhmm "
            + sample_reads + " > /dev/null")
    print cmd
    check_call(cmd, shell=True)
    #Remake oringinal hmmer for this MSA
    domain_class = os.path.splitext(os.path.basename(msa))[0]
    check_call("hmmbuild " + OUTDIR + domain_class + ".hmm " + msa + " > /dev/null"
      , shell=True)
```

Summarise results

We now collect the results and take the best hit of each read to the HMMER model database as it's classification. We can then compare the accuracy of the classification at the sub-domain, domain and Type level.

```
files <- Sys.glob("./temp_output/*_hmmSearch.txt")
search_data <- do.call(rbind, lapply(files, function(f) fread(paste("grep -v ^# ",
    f, sep = ""))))
colnames(search_data) <- c("target name", "accession_t", "query name", "accession_q",
    "E-value1", "score1", "bias1", "E-value2", "score2", "bias2", "exp", "reg",
    "clu", "ov", "env", "dom", "rep", "inc", "description of target")
search_data <- search_data[order(search_data$`E-value1`, decreasing = FALSE),
    ]
search_data <- search_data[!duplicated(search_data$`target name`), ]
search_data$domain <- str_split_fixed(search_data$`target name`, "_", 3)[, 2]
search_data$hmmerHit <- gsub("MA_", "", search_data$`query name`)
search_data$hmmerHit <- gsub("MSA", "", search_data$hmmerHit)

search_data$matchSubDomain <- search_data$domain == search_data$hmmerHit
search_data$matchDomain <- gsub("\\..*", "", search_data$domain) == gsub("\\..*",
    "", search_data$hmmerHit)
search_data$domain_name <- gsub("\\..*", "", search_data$domain)
search_data$type[grepl("DBLa1", search_data$domain_name)] <- "A"
search_data$type[!grepl("DBLa1", search_data$domain_name)] <- "BC"

search_data$typeInferred[grepl("DBLa1", search_data$hmmerHit)] <- "A"
search_data$typeInferred[!grepl("DBLa1", search_data$hmmerHit)] <- "BC"

search_data$typeMatch <- search_data$typeInferred == search_data$type

subdomain_summary <- search_data %>% group_by(domain) %>% summarise(count = n(),
    match = sum(matchSubDomain), accuracy = sum(matchSubDomain)/n())

domain_summary <- search_data %>% group_by(domain_name) %>% summarise(count = n(),
    match = sum(matchDomain), accuracy = sum(matchDomain)/n())

type_summary <- search_data %>% group_by(type) %>% summarise(count = n(), match = sum(typeMatch),
    accuracy = sum(typeMatch)/n())
```

Plot results

Sub-domain level

Although we do not make use of the sub-domain classification it is included here for interest sake. As the sub-domains are often very similar the accuracy in classification with short reads is lower being 60.6% on average. This is low in part because some domain classes have very few domains from which to build the HMMER model. Consequently, when removing a domain in the cross validation procedure we can have a large impact on its respective HMMER model.

```
gg <- ggplot(subdomain_summary, aes(x = domain, y = accuracy)) + geom_col()
gg <- gg + theme_bw()
```

```
gg <- gg + theme(axis.text.x = element_text(angle = 90, hjust = 1, vjust = 0.5))
gg
```

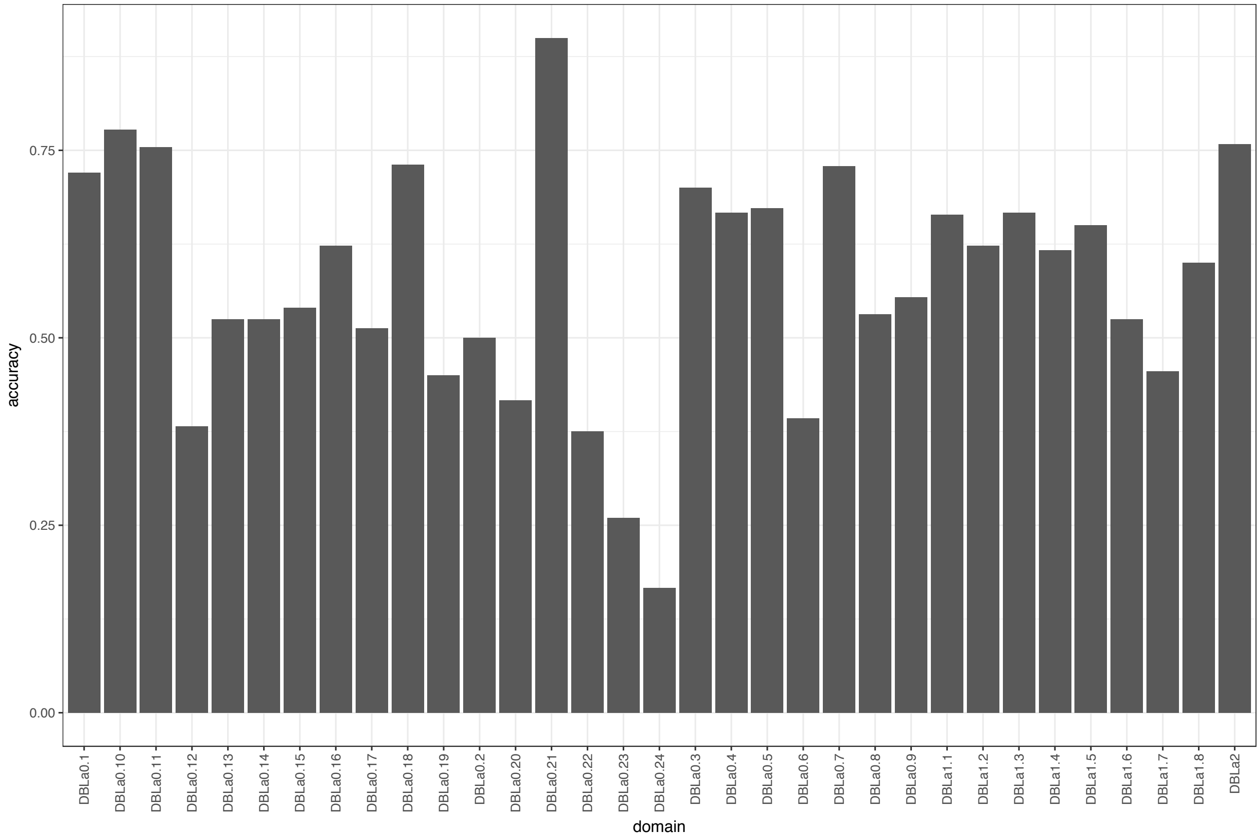

Domain level

At the higher domain levels (DBLa0, 1 and 2) the accuracy is much better due to the greater differentiation between the domain classes giving an accuracy of 99.3%, 98.6% and 75.8% respectively.

```
gg <- ggplot(domain_summary, aes(x = domain_name, y = accuracy)) + geom_col()
gg <- gg + theme_bw()
gg <- gg + theme(axis.text.x = element_text(angle = 90, hjust = 1, vjust = 0.5))
gg <- gg + xlab("domain")
gg
```

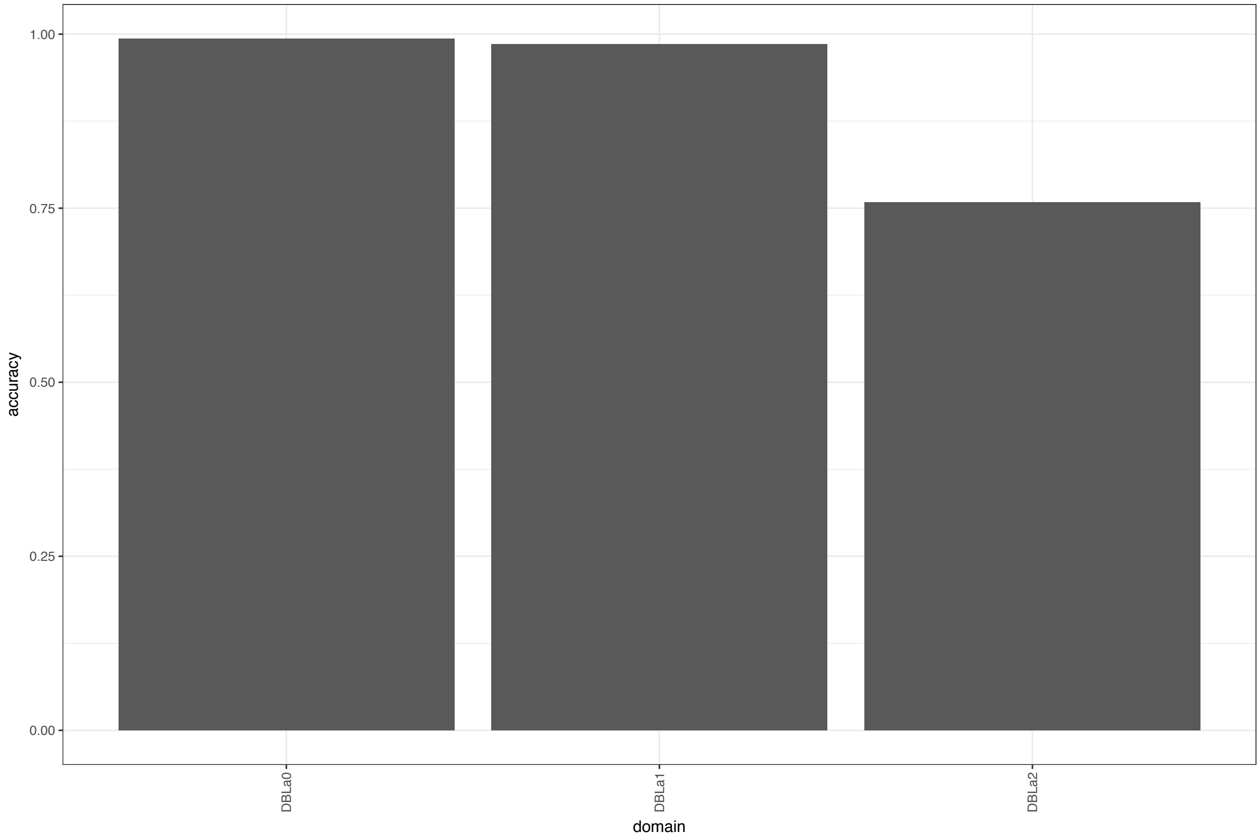

Type level

From the supplementary figure 5 of Rask et al 2010 it can be seen that 4/169 DBLa1 occurrences occurred in UPS type A var genes. Whilst DBLa0 and DBLa2 types occur only in UPS types B and C. From the DBLa sequence it is not possible to distinguish the B and C types. After collapsing the reads into two categories (A and BC) we can see that the classification accuracy is 98.6% and 99.8% respectively.

```
gg <- ggplot(type_summary, aes(x = type, y = accuracy)) + geom_col()
gg <- gg + theme_bw()
gg <- gg + theme(axis.text.x = element_text(angle = 90, hjust = 1, vjust = 0.5))
gg <- gg + xlab("domain")
gg
```

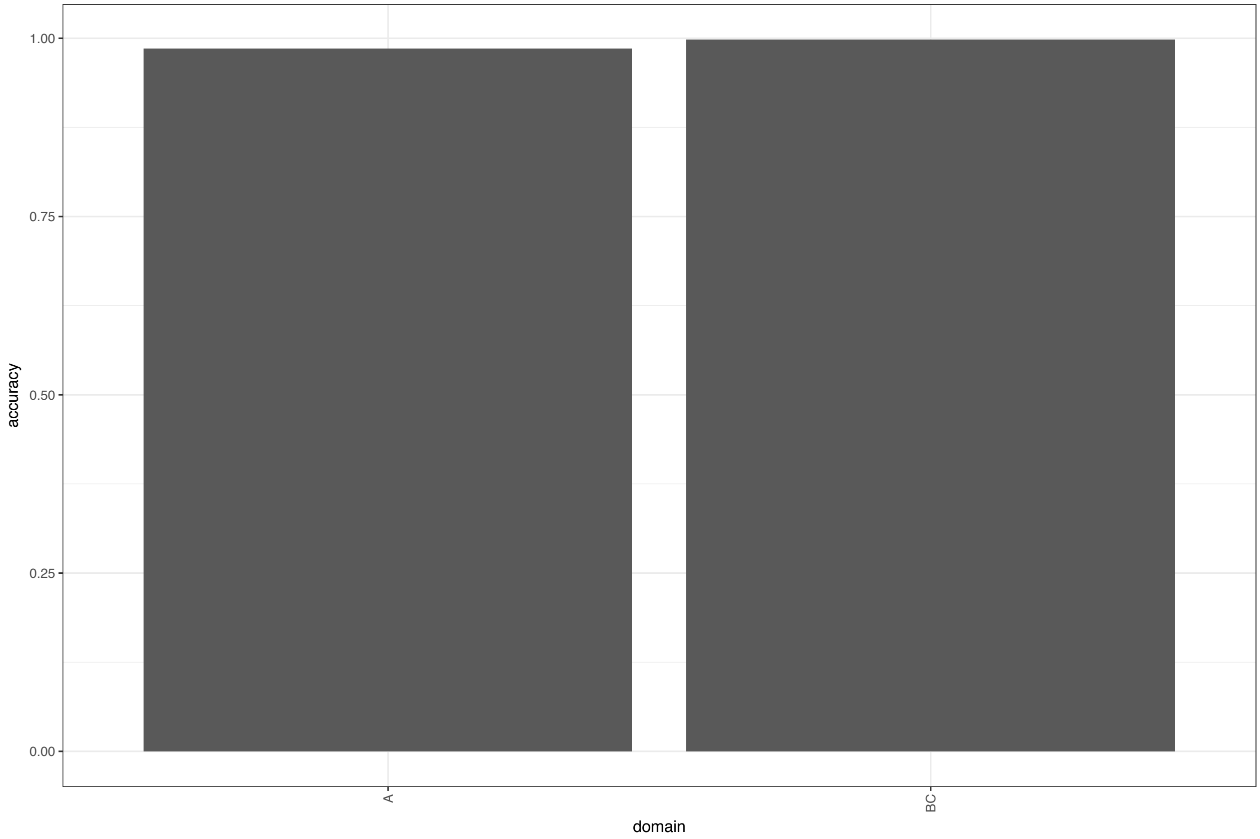

If we assume that 4/169 of the DBLa classifications are incorrect as not all DBLa1 tags are associated with Type A this reduces the Type A classification accuracy down to 96.2%.

Session Information

```
sessionInfo()
```

```
## R version 3.4.1 (2017-06-30)
## Platform: x86_64-apple-darwin15.6.0 (64-bit)
## Running under: OS X El Capitan 10.11.4
##
## Matrix products: default
## BLAS: /Library/Frameworks/R.framework/Versions/3.4/Resources/lib/libRblas.0.dylib
## LAPACK: /Library/Frameworks/R.framework/Versions/3.4/Resources/lib/libRlapack.dylib
##
## locale:
## [1] en_AU.UTF-8/en_AU.UTF-8/en_AU.UTF-8/C/en_AU.UTF-8/en_AU.UTF-8
##
## attached base packages:
## [1] stats     graphics  grDevices utils     datasets  methods   base
##
## other attached packages:
## [1] bindrcpp_0.2      dplyr_0.7.2       stringr_1.2.0     ggplot2_2.2.1
## [5] data.table_1.10.4
##
## loaded via a namespace (and not attached):
```

```
##  [1] Rcpp_0.12.12     bindr_0.1        knitr_1.16       magrittr_1.5    
##  [5] munsell_0.4.3    colorspace_1.3-2 R6_2.2.2         rlang_0.1.1     
##  [9] plyr_1.8.4       tools_3.4.1      grid_3.4.1       gtable_0.2.0    
## [13] htmltools_0.3.6  assertthat_0.2.0 yaml_2.1.14      lazyeval_0.2.0  
## [17] rprojroot_1.2    digest_0.6.12    tibble_1.3.3     formatR_1.5     
## [21] glue_1.1.1       evaluate_0.10.1  rmarkdown_1.6    labeling_0.3    
## [25] stringi_1.1.5    compiler_3.4.1   scales_0.4.1     backports_1.1.0 
## [29] pkgconfig_2.0.1
```
